# Organ Donation After Oral Ingestion of a Voluntary Assisted Dying Substance

**DOI:** 10.5694/mja2.70193

**Published:** 2026-04-22

**Authors:** Joanne Kantianis, Helen I. Opdam, Rohit L. D'Costa

**Affiliations:** ^1^ University Hospital Geelong Geelong Victoria Australia; ^2^ Austin Health Melbourne Victoria Australia; ^3^ Australian Organ and Tissue Authority Canberra Australian Capital Territory Australia; ^4^ Royal Melbourne Hospital Melbourne Victoria Australia

**Keywords:** death, ethics, euthanasia, motor neuron disease, tissue and organ procurement, transplantation

## Abstract

Organ donation after voluntary assisted dying (VAD) is increasingly undertaken in jurisdictions where it is legally permissible, including Australia, although previously all reported cases involved intravenous administration of the life‐ending substance. A 55‐year‐old woman in Victoria has become the first known person to have successfully donated organs and tissues after self‐administration of an oral substance, despite initial uncertainty about feasibility due to the unpredictable time to death (agonal phase). With Victorian legislation requiring self‐administration as the default, this case provides a precedent, opening the possibility of donation for others in this majority VAD cohort where oral administration is obligatory.

## 1 Introduction

Deceased organ and tissue donation is facilitated in individuals who have been determined dead through either neurological criteria (‘brain death’) or where circulatory cessation has led to death (‘circulatory death’). Donation after voluntary assisted dying (VAD) represents a subset of donation following circulatory determination of death (DCDD), and has been undertaken in Australia since 2023. The first organ donor after VAD in Australia was a woman in the State of Victoria who accessed VAD in the context of a terminal motor neuron disease diagnosis [1]. In Australia, the term VAD is applied regardless of whether someone is self‐administering an oral life‐ending substance (assisted suicide) or whether a practitioner is engaged to administer an intravenous life‐ending substance (voluntary euthanasia). Most Australian jurisdictions permitting VAD allow the patient and/or their practitioner some scope in selecting the preferred administration option [2], but Victorian [3] (and South Australian) [4] legislation mandates oral self‐administration unless the person is physically incapable of self‐administration or digestion. In Victoria, only about 15% of VAD permits issued are for intravenous practitioner administration [5]. This is in contrast to the international experience, such as in Canada [6], Spain [7], the Netherlands [8] and Belgium [9], where most assisted deaths occur following intravenously administered voluntary euthanasia. Although VAD medication protocols have not been made public in Victoria, they are reported to contain sedative and other agents used in medical practice [10]; for example, in other countries where data are available, either high dose barbiturates (for oral ingestion) or a combination of opioid, sedative and neuromuscular blockade drugs (for intravenous administration) are typical [11].

Despite growing experience with organ and tissue donation following assisted dying, particularly in the aforementioned countries [6, 12, 13], with satisfactory transplant outcomes reported [14], all cases have been facilitated following intravenous administration of the substance causing death. This is understandable, given that practices overwhelmingly favour intravenous administration in those countries. In addition, a perceived advantage in terms of solid organ donation with the intravenous mode is predictability with respect to a short agonal period—the interval from drug administration to circulatory arrest [14]. In Quebec, Canada, for example, the mean agonal period reported in a series of patients undergoing organ donation after assisted dying was 12.6 min [15]. Evidence in DCDD supports superior outcomes with shorter agonal phases, particularly in terms of liver [16] and heart [17] transplantation. Feasibility for organ donation after VAD in Australia has been considered mainly in the context of intravenous administration [18], with the internal operational guidelines at DonateLife Victoria—the State's organ and tissue donation agency—reflecting this assumption. Further, to minimise the time from death determination to the organ donation surgical procedure, namely the asystolic phase (which follows the agonal phase), the guidelines require that death occurs in a hospital setting, in proximity to the operation theatre.

## 2 Patient KD

KD was a 55‐year‐old woman who had been diagnosed with motor neuron disease (MND) in the context of a rapid decline in limb strength coupled with respiratory muscle weakness and pain. She had been previously well, with her only past medical history being a hernia repair, cervical polyps and previous cigarette smoking. As her disease advanced, KD was assessed and approved for a self‐administration VAD permit. Before her death, KD and her family gave verbal permission for us to report her experience. Following her death, KD's next of kin provided written consent for publication.

Throughout her disease trajectory, KD valued her autonomy and proactive engagement in her care and end‐of‐life decision‐making. Her desire was to be an organ and tissue donor after death, but she reported being advised by multiple healthcare professionals that organ donation following oral VAD was not possible. Nevertheless, she remained determined to pursue donation and independently contacted DonateLife Victoria.

By the time of her initial meeting with donation specialist nurses from DonateLife Victoria, KD had already undertaken extensive end‐of‐life and terminal care planning. This included pre‐booking a limousine to transport her to hospital for the purposes of VAD and potential organ donation. She articulated a clear and informed desire to become an organ donor, supported by substantial personal enquiry.

The preparation for organ donation occurred over several subsequent encounters and included formal consent, a comprehensive medical and social history questionnaire and assessment, a chest x‐ray, a computed tomography (CT) scan of the chest and abdomen, blood investigations and tissue typing. The CT scan identified a lobulated structure in the gastric fundus, raising concern for a gastrointestinal stromal tumour or diverticulum. This finding was reviewed by the transplant surgical teams, and KD consented to a post‐mortem intraoperative biopsy to further assess the lesion. Given familiarity with organ donation procedures, the intensive care unit (ICU) of the local hospital was agreed upon as the location where KD would ingest her VAD medication.

On the day of her choosing, KD was admitted to the ICU at 12:30 h, accompanied by her daughters, friends and her two poodles. An intravenous cannula was placed for heparin administration, and non‐invasive observations and blood tests were taken, along with a swab to test for SARS‐CoV‐2 infection. Blood plethysmography and electrocardiogram (ECG) monitoring was applied. Arterial line placement for invasive blood pressure monitoring was not routine in organ donors after VAD at the time, although this has now been incorporated into the DonateLife Victoria guidelines. At 14.20 h KD ingested the VAD substances and shortly after became unconscious. Her oxygen saturation decreased below 50% at 14:30 h, and there was loss of electrical activity on the ECG trace at 14:54 h. Following a 5‐min observation period of persistent circulatory arrest, death was determined, a total of 39 min following ingestion of the VAD substance. At donation surgery, the gastric fundus was carefully examined and no lesion was identified. Both kidneys, lungs and cardiovascular valvular tissue were retrieved and donated for transplantation, in addition to eye tissue for MND research. The liver was deemed unsuitable for retrieval due to prolonged time to death, resulting in extended warm ischaemia beyond acceptable transplantation thresholds. Whole heart and pancreas were deemed not suitable for transplant due to the combination of the donor's age and the expected agonal phase duration. At routine follow‐up with transplant units, the transplanted recipients were recovering well.

## 3 Discussion

This case is, to our knowledge, the first reported instance of successful organ donation following assisted death from self‐administration of an oral substance. Although KD did not meet the legal requirements to be granted a permit for intravenous practitioner‐administered VAD, this did not present an eventual barrier to work‐up and facilitation of organ and tissue donation. Organ donation after VAD requires first‐person consent from a person who is provided with specific and detailed information about the donation and transplantation process at a time approaching their death. In this sense, it is the strongest embodiment of the principle of autonomous decision‐making in organ and tissue donation, arguably beyond what is possible with first‐person authorisation or donor designation via registries [19]. The number of people accessing VAD in Victoria is small but increasing [3]. By comparison, around 5% of deaths in Canada occur following medical assistance in dying (MAID) [6] and, in the Canadian province of Quebec, 14% of all deceased donors donated after MAID in 2022 [15]. With oral self‐administration being the default for VAD in Victoria, this case has demonstrated that organ donation is feasible and can be realistically considered in this large sub‐group, which comprises the majority of individuals accessing VAD in the state.

However, uncertainties need to be acknowledged. First, the potential contribution of this pathway to the deceased donor pool is unclear. It may be that individuals seeking VAD who are more likely to be medically suitable to donate organs and tissues are also more likely to be granted a practitioner administration permit (e.g., due to a neurological condition that impairs swallowing). Second, although donation for transplantation occurred and early graft function was acceptable, a single case does not demonstrate outcome efficacy, particularly relative to donors after intravenous VAD administration. An important concern would be the potential for a longer agonal period. In KD's case, this period was 34 min, compared with 12.6 min being the mean reported in the Canadian series of donors following intravenous administration. Invasive blood pressure monitoring is routine in DCDD and allows for earlier ascertainment of loss of circulation compared with inactivity on an ECG trace, with, on occasion, electrical cardiac activity persisting beyond mechanical asystole, we have now incorporated this into the DonateLife Victoria donation after VAD guidelines. This case illustrates how, with careful consideration, planning and coordination among VAD clinicians, donation staff and transplant teams have supported an individual in enabling both their right to access VAD as well as their intention to be an organ and tissue donor. It also highlights how many of the principles underpinning VAD as articulated within the Victorian legislation [3]—that is, personal autonomy, informed decision‐making, genuine choice, respect and protection of the vulnerable—have been equally central to the organ and tissue donation process for our patient.

## Author Contributions

**Joanne Kantianis:** writing (original draft), writing (review and editing). **Rohit L. D'Costa:** writing (original draft), writing (review and editing). **Helen I. Opdam:** writing (review and editing).

## Funding

The authors have nothing to report.

## Disclosure

Not commissioned; externally peer reviewed.

## Consent

The patient's next of kin provided written consent for publication.

## Conflicts of Interest

The authors declare no conflicts of interest.

## Data Availability

This article includes no original data.
